# Calculation of Antimicrobial Use Indicators in Beef Feedlots—Effects of Choice of Metric and Standardized Values

**DOI:** 10.3389/fvets.2019.00330

**Published:** 2019-10-09

**Authors:** Stephanie A. Brault, Sherry J. Hannon, Sheryl P. Gow, Simon J. G. Otto, Calvin W. Booker, Paul S. Morley

**Affiliations:** ^1^Department of Clinical Sciences, College of Veterinary Medicine & Biological Sciences, Colorado State University, Fort Collins, CO, United States; ^2^Feedlot Health Management Services Ltd, Okotoks, AB, Canada; ^3^Canadian Integrated Program for Antimicrobial Resistance Surveillance, Public Health Agency Canada, Saskatoon, SK, Canada; ^4^School of Public Health, University of Alberta, Edmonton, AB, Canada; ^5^VERO - Veterinary Education, Research & Outreach Center, Texas A&M University, West Texas A&M University, Canyon, TX, United States

**Keywords:** cattle, animal daily dose, quantification, comparison, duration of effect

## Abstract

The potential for antimicrobial use (AMU) to lead to the development of antimicrobial resistant bacteria is an increasingly important priority in human and veterinary medicine. Accurate AMU quantification is essential to assessing the risk of antimicrobial resistance due to AMU. The quantification of AMU in production animals can be difficult, and feedlot beef cattle present a number of unique challenges. This paper presents selected parenteral data from western Canadian beef feedlots to illustrate variations in interpretation of AMU that can arise from the use of different metrics and standards. Specific examples presented compare the number of animal daily doses calculated from a given amount of antimicrobial drug (AMD) using actual and estimated weights of cattle at exposure, dose-based to weight-based indicators representing the same amount of antimicrobial, dose-based AMU indicators using different estimated durations of effect (DOE), and AMU indicators calculated using different standard weights of cattle at exposure. Changing these factors when calculating AMU indicators can have notable influences on the results obtained. Transparency about the methods used to calculate AMU indicators is critical to ensure that comparisons of use among different populations is meaningful and accurate.

## Introduction

The potential for antimicrobial use (AMU) to promote selection of antimicrobial resistant bacteria is a subject of increasing priority to stakeholders in public and animal health, policy making and international trade (1). In particular, AMU in food-producing animals is under intensifying scrutiny because of potential public health risks putatively associated with contamination of the environment and food products with resistant bacteria (2–4) and direct transmission of resistant bacteria (5). However, the collection and analysis of AMU data in production livestock can be logistically challenging for a number of reasons, and these difficulties have been repeatedly identified as a barrier to understanding AMU and resistance in this context (6, 7). Nonetheless, information about AMU in food-producing animals is critically important for assessing relationships between AMU and antimicrobial resistance (AMR), to understand variability of AMU among different populations, to design or improve AMU monitoring systems, and to inform antimicrobial stewardship efforts.

Antimicrobial drugs are regularly used in North America to maintain feedlot cattle health (8). Almost 90% of feedlots with more than 1,000 head of cattle in the United States reported administering AMDs to cattle by injection or in feed or water in a survey administered in 2016 (9). Bovine respiratory disease (10) and control of liver abscesses (11) are two common therapeutic indications for AMU in feedlot cattle. Use of antimicrobials for production purposes (e.g., growth promotion and feed efficiency) is now limited in Canada and the United States to non-medically important antimicrobials, such as ionophores, and is restricted in Mexico (12–14).

Attempting to quantify AMU in beef cattle is accompanied by many unique challenges in comparison with other production animals. While other species, such as poultry, have a consistent and short production cycle, the life-span, and the related length of time that cattle are intensively managed for finishing is relatively long and can vary significantly. Most cattle raised for beef production in North America are harvested when live bodyweights range from 500 to 640 kg (about 1,100–1,400 lb), and when cattle are typically <2 years of age. The feeding period (the time spent in a feedlot or intensive finishing operation) of beef cattle can vary depending upon weight at placement, feeding conditions, and whether a leaner or fatter animal at finish is desired, but is generally from 90 to 300 days (15). Because of the lengthy feeding period and the relatively large size of cattle at harvest, differences in weight gained during the feeding period can vary by several hundred kilograms. Due to common feedlot practices wherein animals are re-sorted with some frequency to maintain pens of uniformly sized cattle to facilitate feeding and marketing, it can also be difficult to follow individual animals and pen groups because the animal units that make up the pens can change. Finally, while not a problem unique to estimating AMU in feedlot cattle, the duration of effect (DOE) of some antimicrobial drugs (AMD) has not been internationally established (31).

There are many approaches to the quantification of AMU, each with their own unique advantages and disadvantages; no single method is considered to be ideal in all circumstances (16). Measurements used to quantify AMU typically include a numerator describing the amount of AMD animals received and a denominator intended to normalize the numerator by animal weight or the at-risk number of animals or animal-days (7). Taken together, the numerator and denominator are defined as “indicators” of AMU (17). Mass of active ingredient (mg or kg) is intuitive and easily understood as a numerator, especially by lay people. However, this type of measurement can be misleading and inaccurate because it does not account for variations in the mg/kg dosage of antimicrobials (18). Accordingly, dose-based metrics have been adopted by many research groups (19, 20). Dose-based metrics have the advantage of accounting for differences among drugs in concentration and DOE, thus providing an AMU measurement with a more realistic interpretation of the relative contribution of different antimicrobials than weight or count-based metrics (18). In this system, a defined daily dose (average of the range of dosages in units of mg/kg animal/day) must be described for the population of interest for each drug, age-group, and species (21). Employing the defined daily dose and a standard animal weight at exposure, the number of animal daily doses of a particular AMD contained in a given amount of that AMD can be estimated. The selected standard weight selected can have a significant effect on calculated dose-based metrics and should be clearly stated for optimal data interpretation. The standard weight is at best always an approximation, but knowledge of the weight used makes it possible to recalculate metrics for different purposes using other potentially relevant weights (18).

It should be noted that confusion can arise in dose-based data interpretation because terminology has not been uniformly applied throughout the literature and with different methodologies; it is important to recognize these discrepancies when assessing published AMU data and calculations. The World Health Organization^1^ defines the defined daily dose (DDD) in people as “the assumed average maintenance dose per day for a drug used for its main indication in adults;” the DDD as applied in human medicine therefore has mg/day units. In contrast, the European Surveillance of Veterinary Antimicrobial Consumption (ESVAC) group uses the terminology DDD_vet_ to refer to the mg/kg/day dosages for AMDs in different species; it is recommended that the specific term DDD_vet_ be reserved for ESVAC use to avoid confusion (22). Similarly, terminology like “animal daily dose” (ADD) has been used in the literature both to refer to the mg/kg/day dosage (23, 24) and mg/day dosage for an animal of standard weight (18, 25). Since there is such a range in terminology, it is important to clearly state the units associated with each metric reported (26).

Selection of an appropriate denominator to quantify the population of at-risk animals is critical to data interpretation, especially when disparate animal populations are compared. For example, if AMU in mg on a poultry flock was compared to use in mg in a herd of the same number of cattle without normalization, it would seem that the antimicrobial amounts used in the cattle were relatively high compared to the poultry if the larger size of cattle (necessitating a larger dose of AMD per animal) was not considered. For description of AMU at the farm level, ESVAC suggests as a denominator the number of animals present “in a certain weight group or production type and the time present” (27). Dose-based numerators are frequently reported with the denominator of number of exposed animals of a standard weight (28, 29). Number of animal-days has also been advocated as a denominator (17). Denmark reports AMU in different species production classes as the estimated treatment proportion, or the number of defined animal daily doses for each antimicrobial agent by species (mg/kg/day) divided by 1,000 standard animals per day (30).

For national reports of antimicrobial sales data, the total body weight of the animal population is recommended by ESVAC as a denominator (27). The population correction unit (PCU), as defined by ESVAC, is an example of a theoretical estimate of exposed biomass. It is calculated by multiplying the number of animals slaughtered during a given period of time by the standard weight of the animals at the time of exposure (31); the national PCU is then obtained by summing PCU from all sectors of food animal production. The PCU term can be useful as a denominator because 1 PCU is equal to 1 kg of any category of exposed animal and is thus interchangeable among different species, and the overall mg (AMD) per PCU indicator is frequently employed in national reports of veterinary antimicrobial sales data in European countries (19, 32). The Public Health Agency Canada (PHAC) and the U.S. Food and Drug Administration (FDA) are both currently evaluating methods to calculate accurate biomass denominators in their respective countries. Standard weights in use by ESVAC are currently employed by PHAC for calculations of PCU until Canadian estimates can be determined (33). The FDA has recently proposed calculation of a biomass denominator for each of the four major food producing species in the U.S. (cattle, swine, chickens, and turkeys), which would be referred to as a target animal biomass (TAB). The TAB would be calculated by multiplying the estimated number of animals in each group in the U.S. by the average weight at slaughter in kg. Antimicrobial sales data would be reported as the sum in mg of all AMDs for a given target animal species, divided by the species-specific TAB (34). The choice of the standard weight used, as well as adjustments made for animal lifespan, can markedly affect PCU calculations (35), and estimates used for these computations should be clearly stated.

The aim of this publication is to present selected parenteral data from a study of AMU in 36 beef feedlots in western Canada (36) to demonstrate the influence of factors such as cattle weight estimates, choice of indicator, and estimates of DOE of AMD on the calculations of AMU indicators. Four examples will be explored: (1) comparison of dose-based AMU metrics (numerator only) using actual and estimated weights of cattle at exposure; (2) comparison of dose-based to weight-based (e.g., mg of AMD) AMU indicators; (3) comparison of dose-based AMU indicators using different estimated DOE; and (4) comparison of AMU indicators calculated using different standard weights.

## Materials and Methods

The AMU data used for examples in this publication were collected from mixed-breed cattle (*n* = 2,615,082) arriving onto 36 western Canadian feedlots from November 1, 2008 to October 31, 2012, and are comprehensively summarized with descriptive statistics in a separate report (36). Briefly, information about individually administered (parenteral and bolus-dosed) and in-feed AMU for therapeutic and production purposes was recorded from placement until animal exit (death or shipping for slaughter). Data collected for individually administered AMU included unique identification number of the exposed animal, date, animal weight at time of administration, active AMD ingredient, dosage, route, reason for administration (metaphylactic or treatment), and disease/syndrome. Data collected for in-feed AMU was less comprehensive and included the production lot of the exposed cattle, feed delivery date, number of animals in the production lot each day, and number of animals receiving each type of in-feed AMD. Based on their date of arrival into the feedlot, the cattle were divided into 4 placement cohorts (PC1, PC2, PC3, and PC4). Cattle arriving between November 1, 2008 and October 31, 2009 comprised placement cohort 1 (PC1), PC2 comprised cattle arriving between November 1, 2009 and October 31, 2010, PC3 comprised cattle arriving between November 1, 2010 and October 31, 2011, and PC4 comprised cattle arriving between November 1, 2011 and October 31, 2012. While cattle were owned and managed by multiple individuals and companies, their healthcare was overseen by a single veterinary practice (Feedlot Health Management Services Ltd, Okotoks, Alberta; Feedlot Health). Data were summarized and metrics/indicators calculated using SAS® software (Windows version 9.4, SAS Institute, Cary, North Carolina).

For the purposes of this study, the resolution of the parenteral data was superior to the in-feed data collected in that individual animal identification, actual dose of AMD used, and the weight of animal at exposure (in nearly all cases) were recorded for parenteral data and not in-feed data. Therefore, to clearly illustrate the influence of changing various factors on AMU calculations using the same comprehensive data, comparative analyses for AMU of 3 parenteral AMD (tetracyclines, macrolides, and beta-lactams) will be used for all examples, although the principles described are generally applicable to other parenteral AMD and in-feed AMD. These 3 AMD also tend to be administered at different times in the feeding period which makes them particularly useful for contrast in examples where weight at exposure is important.

In this study, “animal daily dose” (ADD) will be employed as the dose-based AMU metric (18); ADD will refer to the mg/day dosage for an animal of standard weight and ADD_kg_ will designate the mg/kg/day dosage. The number of ADD (nADD) in a given amount of antimicrobial will be calculated using [Equation 1; (25)]. Depending upon the purpose of the calculation, the actual weight of the animal at exposure in kg or a standard weight will be used.

(1) $$\begin{array}{l} nADD=\frac{Qty\;of\;active\;substance\;in\;mg\;administered}{ADD\;\left(mg\;per\;kg\;per\;day\right)\;*\;weight\;\left(kg\right)of\;animal} \end{array}$$

Denominators presented in the examples will include “per 100 cattle-at-risk” and “per kg biomass.” The denominator includes the entire time that an animal is at risk for antimicrobial exposure from placement at the feedlot until exit; in other words, nADD/100 cattle-at-risk indicates the number of daily doses of antimicrobial applied on average to 100 cattle in the population from placement to exit. The kg biomass denominator will be calculated as described for PCU, but PCU-specific terminology will not be used because only 1 species of animal is being described (24):

(2) $$\begin{array}{l} kg\;biomass=Number\;of\;animals\;*\;standard\;weight\;\left(kg\right) \end{array}$$

## Results

### Example 1: Comparison of Calculation of Dose-Based Metric of AMU (Numerator) Using Actual and Estimated Weights of Cattle at Exposure

To determine the effect of the weight of the animal on the calculation of dose-based AMU metrics, the nADD calculated with actual exposed animal weights was compared with the nADD calculated from the same dataset using a uniform standard weight. The daily doses (ADD_kg_) in mg/kg/day in the surveilled feedlots for each parenteral drug to which cattle were exposed were calculated by dividing the administered dose (mg/kg) by the estimated exposure days represented by one standard treatment, or the DOE (16). For simplicity, only use of parenteral macrolides, tetracyclines, and beta-lactams are presented (Table 1).

**Table 1:** Administered dose, estimated duration of effect (DOE), and the calculated animal daily dose in mg/kg/day (ADD_kg_) of selected antimicrobial drugs used parenterally throughout the study.

**Parenteral antimicrobial drug**	**Administered dose (mg/kg)**	**DOE (days)**	**ADD_**kg**_ (mg/kg/day)**
**Macrolides**			
Tulathromycin	2.5	3	0.83
Tilmicosin	10.0	3	3.33
Gamithromycin	6.0	3	2.00
Tildipirosin	4.0	3	1.33
**Tetracyclines**			
Oxytetracycline (100 mg/ml)	6.7	1	6.70
Oxytetracycline (200 mg/ml)	20.0	2	10.00
Oxytetracycline (300 mg/ml)	30.0	3	10.00
**Beta-lactams**			
Ceftiofur hydrochloride or sodium	1.1	1	1.10
Ceftiofur crystalline free acid	6.6	3	2.20
Procaine penicillin	20.0	3	6.67

In this dataset, cattle with recorded individual weights were treated with the parenteral antimicrobials listed in Table 1 2,196,473 times. An ADD was calculated for each antimicrobial administration by multiplying the ADD_kg_ for the antimicrobial by the actual exposure weight (kg) of the treated animal. The recorded mg administered to the animal was then divided by the calculated ADD to yield the nADD. Examples of calculations for three observations are presented in Supplementary Table 1. These calculations were performed individually for each observation and then the nADD were summed by antimicrobial type and divided by the number of actual animals exposed to yield the mean nADD at each administration (Table 2). As would be expected, the mean of the nADD for each administration approximated the DOE for each antimicrobial in days. The nADD for macrolides, tetracyclines, and beta-lactams for the same dataset were then estimated using a standard weight (the mean weight of cattle at time of exposure to any AMD) to calculate the ADD rather than a known weight. The mean cattle weight at exposure to AMDs in this dataset (tylosin administered as part of hormone implants excluded) was 336 kg (standard deviation 98 kg; range 45–909 kg). Calculation of ADD for each antimicrobial type was performed based upon the mean weight estimate of 336 kg at the time of antimicrobial exposure and then used to estimate the nADD comprising the mg of antimicrobials used in the population (Table 3). The ADD_kg_ was multiplied by the mean weight to generate the standard animal daily dose (ADD) for each drug. The total mg of antimicrobial used in the population was divided by the standard ADD to yield the nADD. Variation between the estimated nADD using the mean weight at exposure and the nADD calculated using actual weight at exposure is shown as a percentage change between the two ([estimated nADD – “actual” nADD]/“actual” nADD).

**Table 2:** Number of animal daily doses (nADD) of parenteral antimicrobial drugs based on actual recorded animal weights and actual number of animals exposed.

**Parenteral antimicrobial drug**	**Number of administrations**	**Sum of nADD**	**Mean of nADD of each administration**	**Standard deviation of mean of nADD**
**Macrolides**				
Tulathromycin	620,058	1,869,247	3.01	0.22
Tilmicosin	68,087	214,741	3.15	0.22
Gamithromycin	9,260	28,274	3.05	0.24
Tildipirosin	3,358	9,195	2.74	0.14
All macrolides	700,763	2,121,457		
**Tetracyclines**				
Oxytetracycline (100 mg/ml)	4,321	4,375	1.01	0.05
Oxytetracycline (200 mg/ml)	952,951	1,899,370	1.99	0.11
Oxytetracycline (300 mg/ml)	387,256	1,169,307	3.02	0.23
All tetracyclines	1,344,528	3,073,052		
**Beta-lactams**				
Ceftiofur hydrochloride or sodium	203,671	213,103	1.05	0.05
Ceftiofur crystalline free acid	2,440	7,589	3.11	0.13
Procaine penicillin	520	1,649	3.17	1.16
All beta-lactams	206,631	222,341		
**Sum of antimicrobials**	2,251,922	5,416,850		

**Table 3:** Calculation of number of animal daily doses (nADD) using mean weight at exposure for the entire population compared to calculation of nADD using actual weight at exposure (from Table 2) for different antimicrobial types.

**Parenteral antimicrobial drug**	**ADD_**kg**_ (mg/kg/day)**	**Mean weight (kg)**	**Standard ADD (mg/day)**	**Antimicrobial used (mg)**	**nADD (mean weight)**	**nADD (actual weight)**	**Variation (%)**
**Macrolides**							
Tulathromycin	0.83	336	280.0	400,310,350	1,429,680	1,869,247	−23.5
Tilmicosin	3.33	336	1,120.0	189,139,740	168,875	214,741	−21.4
Gamithromycin	2.00	336	672.0	15,009,300	22,335	28,274	−21.0
Tildipirosin	1.33	336	448.0	3,578,760	7,988	9,195	−13.1
All macrolides					1,628,878	2,121,457	−23.2
**Tetracyclines**							
Oxytetracycline (100 mg/ml)	6.70	336	2,251.2	9,386,200	4,169	4,375	−4.7
Oxytetracycline (200 mg/ml)	10.00	336	3,360.0	6,882,331,720	2,048,313	1,899,370	7.8
Oxytetracycline (300 mg/ml)	10.00	336	3,360.0	3,435,537,840	1,022,482	1,169,307	−12.6
All tetracyclines					3,074,964	3,073,052	0.0
**Beta-lactams**							
Ceftiofur hydrochloride or sodium	1.10	336	369.6	113,460,385	306,982	213,103	44.1
Ceftiofur crystalline free acid	2.20	336	739.2	6,793,000	9,190	7,589	21.1
Procaine penicillin	6.67	336	2,240.0	4,334,700	1,935	1,649	17.4
All beta-lactams					318,107	222,341	43.1
**All antimicrobial drugs**					5,051,948	5,416,850	−7.3%

The use of estimated vs. body weights measured at the time of drug administration influenced the results of the nADD calculation for most AMDs in the analysis, with the overall nADD underestimated by 7.3% when mean weights were used for the calculation. Even greater discrepancies were noted when individual antimicrobial classes were examined. For example, macrolide use was underestimated by 23.2% and beta-lactam use was overestimated by 43.1% when mean weights rather than actual weights were used. To explore this contrast further, the mean weights at time of exposure (specifically for macrolides and beta-lactams) were determined (Supplementary Figure 1). The mean exposure weight for macrolides was 267 kg and for beta-lactams was 484 kg.

To demonstrate the effect of accurately estimated weights on calculation of nADD, the mean exposure weights (for macrolides and beta-lactam) were used to recalculate nADD in Table 4; similar to Table 3. Overall, the variation between the two calculations was markedly decreased by using more specific weights for the antimicrobial classes.

**Table 4:** Use of mean weight at exposure for macrolides and beta-lactams compared to actual weight at exposure to calculate the number of animal daily doses (nADD) of the 2 antimicrobial classes.

**Parenteral antimicrobial drug**	**ADD_**kg**_ (mg/kg/day)**	**Mean weight (kg)**	**Standard ADD (mg/day)**	**Antimicrobial used (mg)**	**nADD (mean weight)**	**nADD (actual weight)**	**Variation (%)**
**Macrolides**							
Tulathromycin	0.83	267	223	400,310,350	1,799,148	1,869,247	−3.8
Tilmicosin	3.33	267	890	189,139,740	212,517	214,741	−1.0
Gamithromycin	2.00	267	534	15,009,300	28,107	28,274	−0.6
Tildipirosin	1.33	267	356	3,578,760	10,053	9,195	9.3
Overall macrolides					2,049,825	2,121,457	−3.4
**Beta-lactams**							
Ceftiofur hydrochloride or sodium	1.10	484	532.4	113,460,385	213,111	213,103	0.0
Ceftiofur crystalline free acid	2.20	484	1,065	6,793,000	6,378	7,589	−15.9
Procaine penicillin	6.67	484	3,227	4,334,700	1,343	1,649	−18.6
Overall beta-lactams					220,832	222,341	−0.68

### Example 2: Comparison of Dose-Based and Weight-Based AMU Indicators

#### Part 1: Comparison of Use of Two Different Antimicrobial Classes

**Figure 1:**
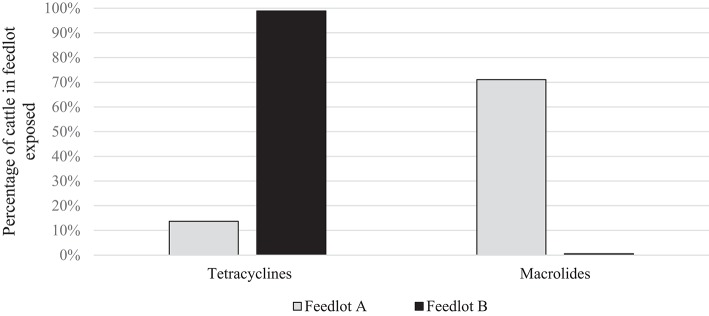
Percentage of cattle exposed to tetracyclines and macrolides parenterally in Feedlots A and B.

To directly compare AMU quantification in different AMD classes (dose-based vs. weight-based indicators), parenteral AMU data for macrolides and tetracyclines from two feedlots (A and B) are presented. These particular feedlots and antimicrobials were compared because of the marked contrast in the proportion of cattle exposed to the two antimicrobial types in the two feedlots, and the relatively lower mg/kg dosage of macrolides; accentuating differences between dose-based and weight-based metrics. As can be seen in Figure 1, the proportion of cattle exposed to tetracyclines parenterally was higher in Feedlot B than A, and the proportion of cattle exposed to macrolides was higher in Feedlot A than B.

The dose-based AMU indicator selected for this example was the nADD per 100 cattle-at-risk. Prerequisite for this calculation was the ADD in mg/kg/day or ADD_kg_ (calculated in Table 1). The ADD [total mg of the particular antimicrobial drug administered to a “standard” animal (mg/day)], was then obtained by multiplying the ADD_kg_ (mg/kg/day) by the mean cattle weight at exposure averaged over both feedlots (kg) (Table 5).

**Table 5:** Parenteral macrolides and tetracyclines used in Feedlots A and B and the calculation of the animal daily dose (ADD).

**Parenteral antimicrobial drug**	**ADD_**kg**_ (mg/kg/day)**	**Mean weight (kg)**	**ADD (mg/day)**
**Macrolides**			
Tulathromycin	0.8	375.6	300.5
Tilmicosin	3.3	375.6	1239.5
**Tetracyclines**			
Oxytetracycline (100 mg/ml)	6.7	375.6	2516.5
Oxytetracycline (200 mg/ml)	10.0	375.6	3756.0
Oxytetracycline (300 mg/ml)	10.0	375.6	3756.0

**Figure 2:**
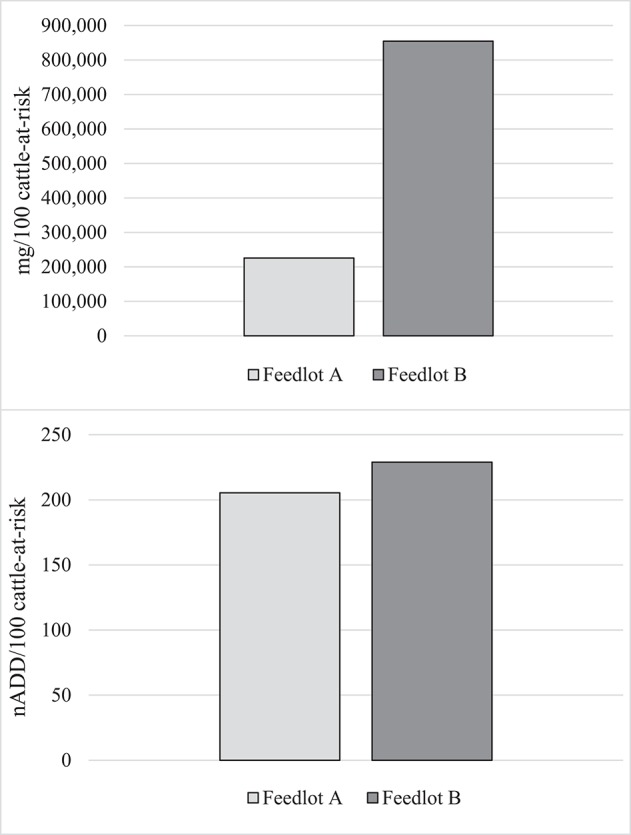
Summed use of parenteral macrolides and tetracyclines in Feedlots A and B in mg antimicrobial drug/100 cattle-at-risk and number of animal daily doses (nADD)/100 cattle-at-risk.

The total mg of macrolides and tetracyclines used in each feedlot were summed from administration records. The total mg amount of each AMD type was then divided by the specific ADD for each drug to yield the nADD consumed in each feedlot, and the summed total mg and the nADD were divided by the number of cattle-at-risk/100 to provide the mg/100 cattle-at-risk and the nADD/100 cattle-at-risk (Table 6). Figure 2 presents AMU of both AMD types, depending upon whether mg AMD or nADD are used in the calculation.

**Table 6:** Calculation of total mg antimicrobial drug/100 cattle-at-risk and number of animal daily doses (nADD)/100 cattle-at-risk for parenteral macrolides and tetracyclines in Feedlots A and B.

	**Total mg**	**ADD (mg/day)**	**nADD**	**Number of cattle**	**Number of cattle/100**	**mg/100 cattle-at-risk**	**nADD/100 cattle-at-risk**
**FEEDLOT A**							
**Macrolides**							
Tulathromycin	36,059,760	300	120,007	103,272	1,033	34,917	116
Tilmicosin	73,202,700	1,239	59,059	103,272	1,033	70,883	57
**Tetracyclines**							
Oxytetracycline (100 mg/ml)	107,600	2,517	43	103,272	1,033	104	0
Oxytetracycline (200 mg/ml)	43,719,800	3,756	11,640	103,272	1,033	42,335	11
Oxytetracycline (300 mg/ml)	80,155,500	3,756	21,341	103,272	1,033	77,616	21
**Macrolides and tetracyclines**	233,245,360		212,090	103,272	1,033	225,855	205
**FEEDLOT B**							
**Macrolides**							
Tulathromycin	2,710,190	300	9,020	582,133	5,821	466	2
Tilmicosin	0	1,239	0	582,133	5,821	0	0
**Tetracyclines**							
Oxytetracycline (100 mg/ml)	950,000	2,517	378	582,133	5,821	163	0
Oxytetracycline (200 mg/ml)	4,105,345,220	3,756	1,093,010	582,133	5,821	705,225	188
Oxytetracycline (300 mg/ml)	865,935,240	3,756	230,547	582,133	5,821	148,752	40
**Macrolides and tetracyclines**	4,974,940,650		1,332,954	582,133	5,821	854,606	230

It can clearly be seen that mg as a measurement of AMU confuses interpretation when different classes of AMD are used at disparate levels in the two populations compared. Macrolide use was more common in Feedlot A, while Feedlot B used proportionately more tetracyclines. Since tetracycline mg/kg dosages tend to be higher than macrolides, this inflated the AMU measurement in Feedlot B compared to Feedlot A when the mg metric was used. When the nADD metric (accounting for differences in concentration between the two AMD classes) was used, AMU measurements between the two feedlots were much closer.

#### Part 2: Intra-Class Comparison of Antimicrobial Drug Use

**Figure 3:**
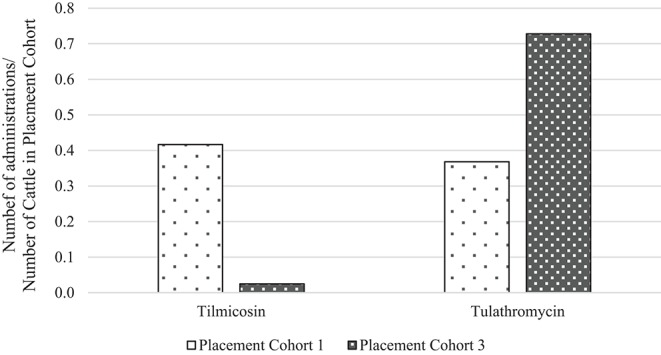
Relative frequency of tilmicosin and tulathromycin use in placement cohorts 1 and 3* of Feedlot A. *Placement cohort 1 comprised cattle placed in feedlot between November 1, 2008 and October 31, 2009, and placement cohort 3 comprised cattle placed in feedlot between November 1, 2010 and October 31, 2011.

**Figure 4:**
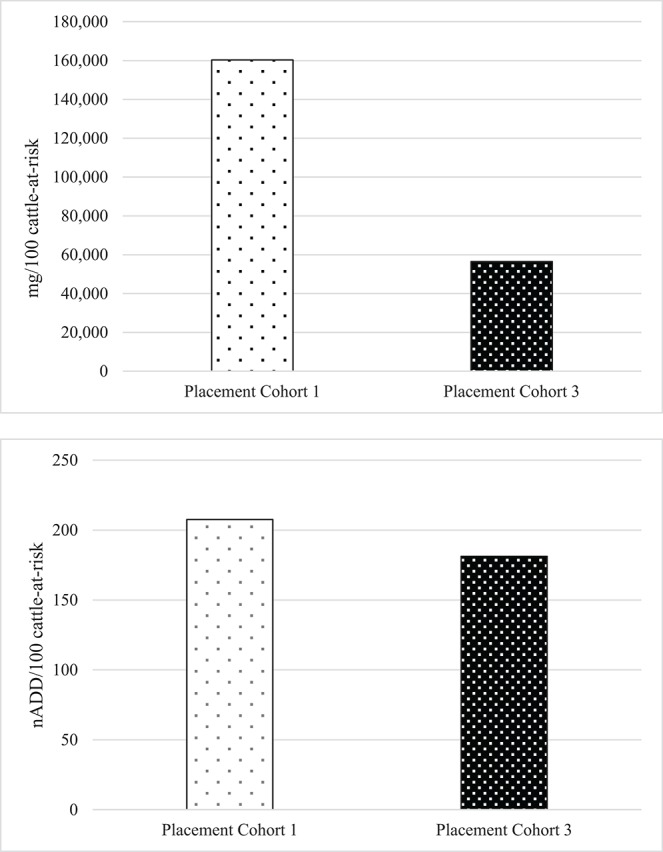
Overall use of macrolides in Feedlot A in mg antimicrobial drug/100 cattle-at-risk and number of animal daily doses (nADD)/100 cattle-at-risk in placement cohorts 1 and 3*. *Placement cohort 1 comprises cattle placed in feedlot between November 1, 2008 and October 31, 2009, and placement cohort 3 comprises cattle placed in feedlot between November 1, 2010 and October 31, 2011.

To directly compare AMU quantification of different types of the same drug class obtained by dose-based vs. weight-based indicators, macrolide use in Feedlot A from two groups of cattle was considered: Cattle entering Feedlot A between November 1, 2008 and October 31, 2009 comprised Placement Cohort 1, and cattle entering Feedlot A between November 1, 2010 and October 31, 2011 comprised Placement Cohort 3. The relative frequency of tilmicosin use decreased while the relative frequency of tulathromycin use increased over time (Figure 3). Use of parenteral macrolides (either tulathromycin 2.5 mg/kg or tilmicosin 10 mg/kg) expressed as mg/100 cattle-at-risk and nADD/100 cattle-at-risk is presented; overall and by type (Table 7, Figure 4). The average cattle weight in Feedlot A (338 kg) and the number of cattle in each placement cohort was used to calculate the kg biomass for each placement cohort.

**Table 7:** Calculation of total mg antimicrobial drug/100 cattle-at-risk and number of animal daily doses (nADD)/100 cattle-at-risk for macrolides in placement cohorts 1 and 3^*^ of Feedlot A.

	**Total mg**	**Mean weight (kg)**	**ADD_**kg**_ (mg/kg/day)**	**ADD (mg/day)**	**nADD**	**Number of cattle**	**Number of cattle/100**	**mg/100 cattle**	**nADD/100 cattle**
**Placement cohort 1**									
Tilmicosin	38,283,600	338	3.3	1,127	33,980	28,200	282	135,757	120
Tulathromycin	6,911,910	338	0.8	282	24,539	28,200	282	24,510	87
All macrolides	45,195,510	338			58,519	28,200	282	160,267	207
**Placement cohort 3**									
Tilmicosin	2,189,100	338	3.3	1,127	1,943	30,339	303	7,215	6
Tulathromycin	14,935,620	338	0.8	282	53,026	30,339	303	49,229	175
All macrolides	17,124,720	338			54,969	30,339	303	56,444	181

* *Placement cohort 1 comprises cattle placed in feedlot between November 1, 2008 and October 31, 2009, and placement cohort 3 comprises cattle placed in feedlot between November 1, 2010 and October 31, 2011*.

Because of the substitution of tulathromycin (lower mg/kg dose) for tilmicosin (higher mg/kg dose) that occurred in Feedlot A over time, macrolide use appears to decrease when the mg/100 cattle-at-risk indicator is used. However, if calculated as nADD/100 cattle-at-risk it can be seen that macrolide use in this feedlot was relatively stable or only slightly decreased over time.

### Example 3: Comparison of Dose-Based Indicators Calculated Using Different Duration of Effect Estimates

**Figure 5:**
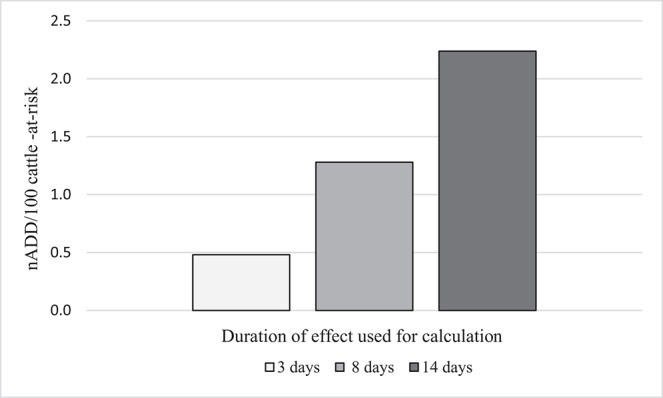
Effect of different durations of effect (DOE) for tulathromycin on the calculation of number of animal daily doses (nADD/100 cattle-at-risk) in Feedlot C.

As has been seen in the previous examples, the ADD_kg_ for each drug is assigned by dividing the administered dose by “the number of days of duration of the therapeutic effect of the substance (22).” However, the length of time an antimicrobial may exert selective pressure on bacteria is not always clear. For tulathromycin, a long acting macrolide frequently administered metaphylactically for BRD, many possible DOE could be proposed based on pharmacokinetic data and expert opinion. Three possibilities are given here: (1) Three days [plasma elimination half-life of the drug (37) and also a standard post-metaphylaxis interval, i.e., the number of days that must elapse before a metaphylactically exposed animal should be treated for BRD] (38). (2) Eight days (elimination half-life in the lung) (37) and value used in daily dose calculation for tulathromycin by ESVAC (22). (3) Fourteen days (estimated DOE in product literature from Zoetis) (39). For illustration purposes, the effect of these 3 different DOE on the nADD/100 cattle-at-risk calculation for one of the feedlots (Feedlot C) is shown (Table 8, Figure 5). Feedlot C contained 178,089 cattle over the course of the study with a mean weight of 291 kg at exposure to tulathromycin.

**Table 8:** Effect of use of three different durations of effect on calculations of number of animal daily doses (nADD)/100 cattle-at-risk for tulathromycin in Feedlot C.

**Duration of effect**	**ADDkg (mg/kg/day)**	**Mean weight (kg)**	**ADD (mg/day)**	**Antimicrobial used (mg)**	**nADD**	**Number of cattle-at-risk**	**Number of cattle-at-risk/100**	**nADD/100 cattle**
3 days	0.83	291	242.1	206,814	854.3	178,089	1,781	0.5
8 days	0.31	291	90.8	206,814	2278.2	178,089	1,781	1.3
14 days	0.18	291	51.9	206,814	3986.8	178,089	1,781	2.2

It can be seen from these data that as the DOE increases, the computed AMU indicator (nADD/100 cattle-at-risk) increased if the number of cattle-at-risk, the mg tulathromycin applied to the population, and the standard cattle weight were held constant. A DOE of 14 days more than quadrupled the calculated indicator of tulathromycin use in this feedlot compared to a DOE of 3 days, demonstrating the importance of DOE choice in the determination of AMU indicators.

### Example 4: Comparison of Weight-Based AMU Indicators Calculated Using Different Biomass at Risk Estimates

**Figure 6:**
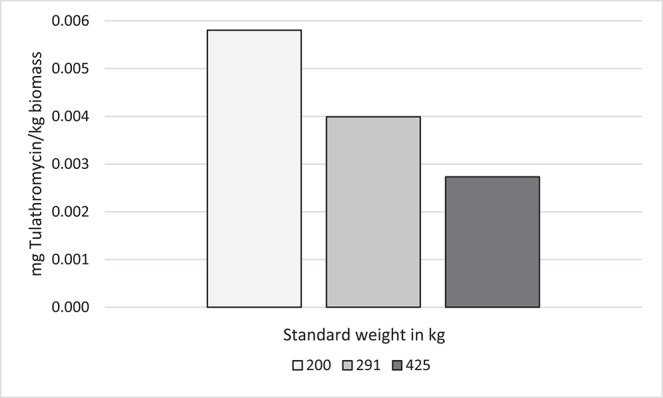
Effect of standard weight on antimicrobial use indicators (mg tulathromycin/kg biomass)* in Feedlot C. *kg biomass = standard weight * number of cattle-at-risk.

To assess the effect of standard weights on AMU indicators using kg biomass as the denominator, 3 different standard weights were applied to data from Feedlot C (from the previous example). Since mg/kg biomass is a commonly reported indicator, mg tulathromycin is presented. Because only one type of AMD is measured by this indicator, no distortion of the calculated values is created by varying AMD concentration and DOE. The standard weights chosen were the known mean exposure weight for tulathromycin (291 kg in Feedlot C), the ESVAC standard heifer weight (200 kg), and the ESVAC standard steer and bullock weight (425 kg) (40). The kg biomass was calculated as previously described by multiplying the number of cattle-at-risk by the standard weight at time of exposure (Supplementary Table 2, Figure 6).

In contrast to DOE, as standard weight increased, the computed AMU indicator (mg/kg biomass) decreased with mg tulathromycin and the number of animals-at-risk held constant. A standard weight of 200 kg approximately doubled the calculated tulathromycin use indicator compared to a standard weight of 425 kg.

## Discussion

The presented examples show that variations in the animal weight or antimicrobial DOE can potentially have a profound effect on calculated AMU indicators. Standardization of measurement is critical whether AMU data will be used for temporal comparisons over time in the same population, benchmarking, or estimation of selective pressure on development of bacterial resistance to antimicrobials (7). The four examples presented in this study each illustrate the influences of particular factors on the calculation of AMU indicators. Example 1 demonstrates that if dose-based metrics such as nADD are to be calculated, it is preferable to use standard weights specific to antimicrobial type for the most accurate results in cases where antimicrobials tend to be administered only at a specific point in the feeding period. Corresponding with the fact that parenteral macrolides are typically administered near the time of arrival at the feedlot, while beta-lactams are administered later in the feeding period, the mean exposure weight for macrolides was much lower than that of beta-lactams. While this type of adjustment has not been routinely done in the calculations of dose-based metrics for production animals such as swine and poultry that have smaller weight increases through the production cycle, it may be particularly important in beef cattle given the large variations in weight at exposure for drugs routinely given at placement vs. drugs given later in the feeding period. Because it is rare to have such detailed information, such as weights of cattle at exposure, as was available in this study, the use of standard weights specific to antimicrobial type may not always be feasible.

However, the potential inaccuracy introduced by the use of one standard weight for all antimicrobials should be recognized. Example 2 demonstrated the advantages of dose-based metrics rather than weight-based metrics if there is variation in the AMD type used by populations that are to be compared. Weight-based metrics, such as mg or kg of active ingredient, are meaningless if AMD with different concentrations and DOE are being compared. In some food animal production systems with minimal variation in AMD type, this may not be as crucial, but in beef cattle, the routine use of both tetracyclines and macrolides, which have significant differences in mg/kg dosage, in herds makes this distinction particularly significant if comparing AMU among herds with disparate prescription practices. Employing weight-based metrics, herds administering more parenteral tetracyclines over macrolides would appear to have heavier AMU than herds administering more parenteral macrolides over tetracyclines, whereas dose-based metrics would tend to indicate the reverse. Consequently, emphasis on weight-based metrics and mg/kg reduction targets could even promote the use of macrolides in preference to tetracyclines, inadvertently encouraging the use of AMD of higher medical importance to humans (41). Comparing national sales data in mg/kg to animal census AMU data available in Denmark and the Netherlands, Bondt et al. (42) concluded that “simple country comparisons, based on total sales figures, entail the risk of serious misinterpretations, especially if expressed in mg per kg.” They noted that to make meaningful international comparisons, the average dosage of the AMD used as well as relative differences in production animal species needed to be taken into account. However, at the national level, the collection of data with enough detail to calculate dose-based metrics is costly and time-consuming, leading most countries to employ the use of aggregated AMD sales data in mg as a proxy for AMU due to resource limitations (43). Recording of more detailed data about AMU by class and species-specific AMD dosages and applications on sentinel farms is recommended when feasible to complement sales data (44). There is ultimately no single AMU metric that is ideal in all situations and a balance must be struck between practicality, accuracy, efficiency, and clarity (16).

For longer-acting antimicrobials such as macrolides, Example 3 illustrated that the choice of DOE in calculating AMU indicators was critical; use of longer DOE resulted in the calculation of higher dose-based use metrics. If benchmarking comparisons are performed, the actual value of the DOE is less important than ensuring that the same one is used for both populations. However, for studies quantifying AMU for the purposes of evaluating influence on bacterial AMR selection, the DOE choice will affect the AMR pressure intensity assigned to an AMD, but data to provide guidance on the correct DOE choice are often lacking. The concentration and persistence of macrolides in lung tissue for up to 14 days may be very important to selection of AMR in *Mannheimia haemolytica* (a bacteria related to BRD often present in the lungs of affected cattle). However, macrolides may not have as prolonged an effect on fecal bacteria such as *Enterococcus* spp. and *Escherichia coli*, bacteria frequently of interest in AMU/AMR studies, so it may not be appropriate to use the same DOE for considering AMR in all bacteria. More data are needed about the DOE of long-acting AMDs in specific compartments of the exposed animal, and their influence on AMR selection in different bacterial species and niches.

Finally, the choice of standardized weight in the calculation of estimated biomass denominators may be very influential on calculated AMU indicators, as demonstrated in Example 4. The FDA and ESVAC currently have differing policies related to standard cattle weights for AMU metrics, with ESVAC specifying that estimated weight at exposure should be used while FDA proposes that average weight at slaughter be used for calculations (27, 34). Since weight at slaughter will almost certainly be significantly higher than weight at exposure for cattle, if the mg/kg biomass for a cattle population calculated by ESVAC conventions were compared to mg/TAB for the same cattle population calculated by FDA conventions, the ESVAC AMU indicator would be higher than the calculated FDA antimicrobial use indicator. Clearly, standardization of animal weight used for calculation of the denominator of AMU indicators is crucial if these data are to be compared internationally, particularly if metrics are to be considered in the context of international trade.

Previous studies have also demonstrated that AMU estimates derived from the same data set can vary depending upon the metric calculated. Mills et al. (21) described the application of 5 different metrics to AMU data in dairy cattle in the United Kingdom (UK): total mg, total mg/kg, daily dose, course dose, and cow calculated course. Similar to Examples 1 and 2 in the present study, these authors concluded that UK-specific AMD dosages and weights should be used for calculation of dose-based metrics and that the mg/kg indicator was only suitable for tracking AMU on a single farm when AMU patterns did not change. Similar to Example 3 in the present study, Taverne et al. (45) found that the use of different country-specific DOE correction factors for long-acting AMD in swine resulted in disparate calculations of dose-based metrics, and recommended harmonization of units of measurement to enable accurate comparisons. These studies presented examples of metric calculations in parenteral, intramammary, and in-feed data, underscoring the applicability of these concepts to AMD given by any route.

The quantification of AMU is increasingly important in both people and animals, and special features of beef cattle introduce additional challenges to an already complex venture. Regardless of the species of interest, consistency of approach (while still tailoring standards as much as possible to the study population) is of paramount importance. Clear definitions, transparent technique communication, and methodology validation are all key to the ability to compare AMU indicators between different populations of animals within species, between species, and internationally.

## Data Availability Statement

The datasets generated for this study are available on request to the corresponding author.

## Ethics Statement

The protocol for this project was reviewed and approved by the Feedlot Health Management Services Ltd. Animal Care Committee (a certified holder of a Certificate of Good Animal Practice) and in accordance with standards set by the Canadian Council of Animal Care.

## Author Contributions

SB drafted the manuscript. SB, SH, SG, SO, CB, and PM were involved in evaluation and interpretation of AMU data, determination of relevant examples, and manuscript revision.

### Conflict of Interest

CB is part owner and managing partner of Feedlot Health Management Services and Southern Alberta Veterinary Services Ltd. SH is an employee of Feedlot Health Management Services Ltd, Okotoks, Alberta, Canada. Feedlot Health is a private company that provides expert consultation regarding production and management of feedlot cattle and calf grower calves, including developing veterinary protocols to support animal health. They also conduct in-house and contract research related to dairy calf grower and feedlot production. The remaining authors declare that the research was conducted in the absence of any commercial or financial relationships that could be construed as a potential conflict of interest.

## Footnotes

^1^World Health Organization. *WHO Collaborating Centre for Drug Statistics Methodology*. World Health Organization. Available online at: https://www.whocc.no/ (accessed April 15, 2018).
