# Supplementary figures and images for: Calculation of Antimicrobial Use Indicators in Beef Feedlots—Effects of Choice of Metric and Standardized Values
Source: Front Vet Sci. 2019 Oct 9;6:330. doi: 10.3389/fvets.2019.00330 (PMC6794351; doi:10.3389/fvets.2019.00330)

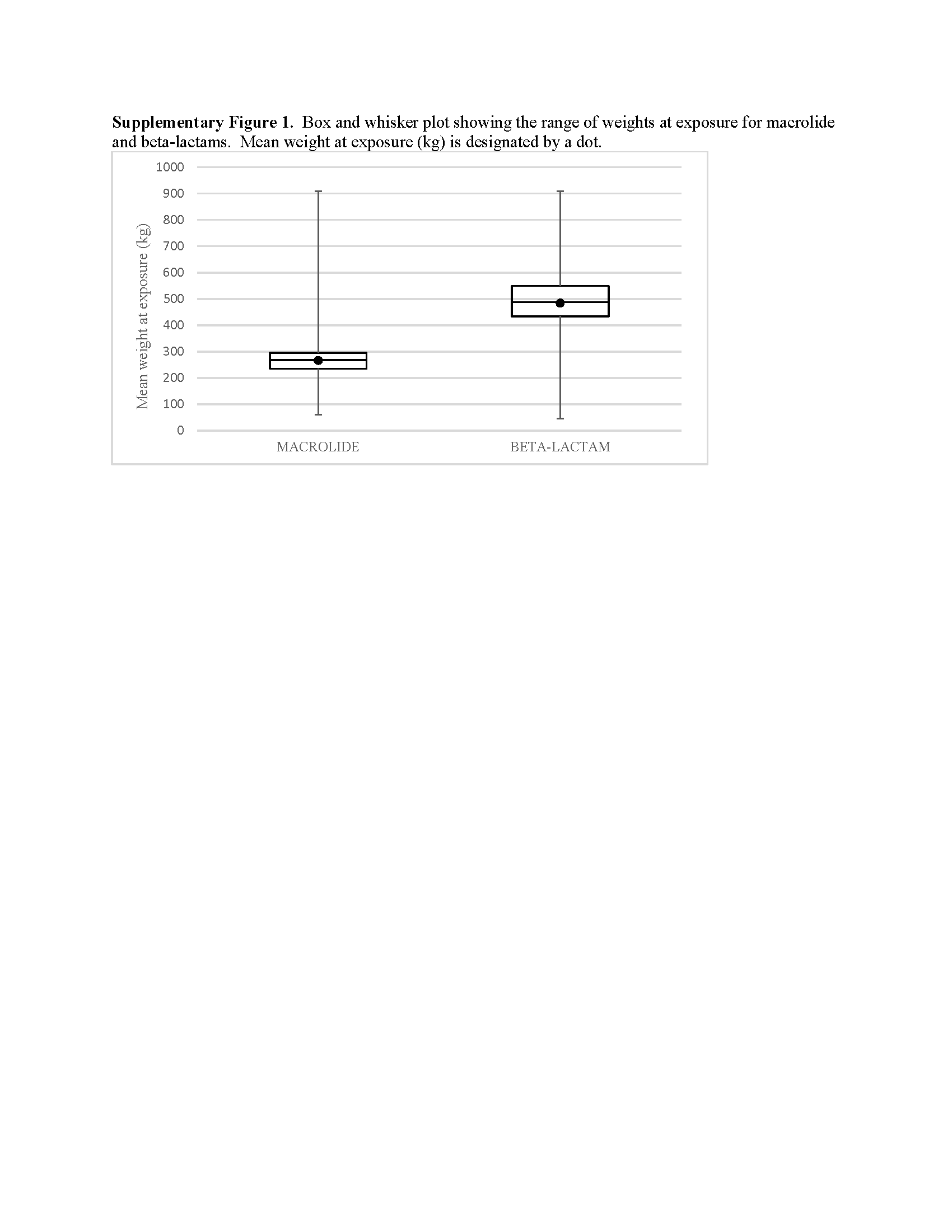

Supplement: Supplementary file 1 [file Image_1.TIFF]
